# Supplementary material for: Bridging the knowledge-practice gap: A cross-sectional survey assessing physician knowledge, attitude and practice toward complementary and alternative medicine
Source: PLoS One. 2025 May 14;20(5):e0322613. doi: 10.1371/journal.pone.0322613 (PMC12077789; doi:10.1371/journal.pone.0322613)
Supplement: S2 File — (DOCX) [file pone.0322613.s002.docx]

S2_Instrument

Domains and items used in the current survey:

| Demographic characteristics | Age (year) |
| --- | --- |
|  | Gender |
|  | Nationality |
|  | Marital Status |
|  | Job level |
|  | Work experience (years) |
|  | Current health facility of work |
|  | Specialty |
| Awareness | Ever heard about complementary medicine |
|  | If yes, the source of information about complementary medicine |
|  | Complementary medicine treatment options you are aware of |
|  | Aware about the harmful effects of complementary medicine |
|  | If yes, list the harmful effect(s) of complementary medicine |
| CAM training | Ever received pre-service training in complementary medicine |
|  | If yes, where do you get it ? |
| Attitudes towards CAM | Agree with the provision of both complementary medicine and modern medicine in combination? |
|  | Providing both complementary medicine and modern medicine for patients could increase patient satisfaction? |
|  | Medical practitioners should be more educated in the use of complementary medicine |
|  | Support the incorporation of complementary medicine in the medical curriculum |
|  | Research about the efficacy and safety of complementary medicine should be performed |
|  | The provision of wellness centers using complementary medicine and modern medicine benefit patients |
| Practice and recommendations to use CAM | Do you have plans to use complementary medicine in the future? |
|  | Do you ask patients about complementary medicine usage? |
|  | Do you recommend the use of complementary medicine for your patients? |
|  | If Yes, what types of complementary medicine do you typically recommend to your patients? |
|  | Reasons to prefer complementary medicine over modern medicine |
|  | Have you used any complementary medicine in the last two years for yourself? |
|  | If yes, what kinds of complementary medicine did you use? |
