# Supplementary material for: Bridging the knowledge-practice gap: A cross-sectional survey assessing physician knowledge, attitude and practice toward complementary and alternative medicine
Source: PLoS One. 2025 May 14;20(5):e0322613. doi: 10.1371/journal.pone.0322613 (PMC12077789; doi:10.1371/journal.pone.0322613)
Supplement: Attachment — Submitted filename: critical review .docx [file pone.0322613.s003.docx]

Thanks for your valuable investigation in terms on CAM. Some points mentioned below:

- The duration of sampling was 3 months which could be wider to earn more participant. Your involved 159 physicians, which might not be representative of the entire physician population in the Jazan region.
- Please give information about validity and reliability of your questionnaire
- The study notes that only 25.8% of physicians plan to use CAM in practice, but it does not explore the barriers to integration in detail. Identifying and addressing these barriers could provide more actionable insights.
- Consider whether the findings can be generalized to other regions or healthcare systems. The cultural and healthcare context of the Jazan region might limit the applicability of the results elsewhere.
- You said that only 25.8% of physicians plan to use CAM in practice, but it does not explore the barriers to integration in detail. Identifying and addressing these barriers could provide more actionable insights.
