# Supplementary material for: Bridging the knowledge-practice gap: A cross-sectional survey assessing physician knowledge, attitude and practice toward complementary and alternative medicine
Source: PLoS One. 2025 May 14;20(5):e0322613. doi: 10.1371/journal.pone.0322613 (PMC12077789; doi:10.1371/journal.pone.0322613)
Supplement: Attachment — Submitted filename: CAM - Manuscript with comments.docx [file pone.0322613.s004.docx]

Bridging the Knowledge-Practice Gap: A Cross-sectional Survey Assessing Physician Knowledge, Attitude and Practice toward Complementary and Alternative Medicine

Sarah M. Salih^1^, Tif A. Jawahi^2^, Rafif H. Al Salem^2^, Shahad A. Alhazmi^2^, Atheer A. Buayti^2^, Arwa H. Alammari^2^, Hadeel M. Mashi^2^, Layla A. Dobea^2^, Mohammed A. Muaddi^1^

^1^ Family and Community Medicine Department, Faculty of Medicine, Jazan University, Jazan City 45142, Saudi Arabia

^2^ Faculty of Medicine, Jazan University, Jazan City 45142, Saudi Arabia

Correspondence*:* [ssalih@jazanu.edu.sa](mailto:ssalih@jazanu.edu.sa)

*Background*

Complementary and alternative medicine (CAM) is widely used in multiple countries, including Saudi Arabia, yet its integration into mainstream healthcare requires evidence-based guidance. However, little is known about the current level of awareness attitudes and practice of physicians regarding CAM in Jazan region. This study assessed physicians’ awareness, attitudes, and practices regarding CAM in the Jazan region.

*Methods*

We conducted a cross-sectional survey among physicians in five governorates of Jazan using convenience sampling. The structured questionnaire explored CAM-related awareness, attitudes, and practices. Data were analyzed using descriptive and inferential statistics to identify associated factors.

*Results*

Of 159 responding physicians (58.5% male), 81.1% were aware of CAM, but only 7.5% had received formal pre-service training. Attitudes towards CAM were generally positive, with a median attitude score of 23/ 30, particularly towards the need for more research and education on CAM. However, only 25.8% planned to use CAM in their practice, and 37.7had recommended it to patients. Factors significantly associated with awareness and practice included age, gender, work experience, and healthcare setting.

*Conclusions*

Despite high awareness and positive attitudes towards CAM among Jazan physicians, there is a significant gap in formal training and limited integration into practice. Our findings highlight the need to formulate targeted educational initiatives and evidence-based guidelines to enhance physicians’ competencies and support the effective implementation of CAM in patient care.

**Keywords:** Complementary and alternative medicine; healthcare; awareness; attitudes; practice

Introduction

Complementary and alternative medicine (CAM) has recently garnered significant attention as a potential adjunct or alternative to medical treatment modalities. CAM is defined as a diverse group of healthcare practices which are not considered a part of conventional medical therapies [1]. These practices include a broad range of modalities, such as acupuncture, herbal medicine, chiropractic care, and homeopathy. Globally, CAM is used on a wide scale, with estimates indicating that 36% of adults in the United States have utilized one or more forms of CAM, and the prevalence is higher in some countries [2]. Early evidence from European countries showed that 20%-50% of individuals have used CAM modalities, with highest estimates in Germany and France (46% and 49%, respectively) [3]. In Australia, approximately 68.9% of the population have reported using CAM, particularly yoga, meditation and chiropractic care [4]. Studies published in Asian countries revealed varied utilization rates of CAM practices, such as traditional Chinese medicine, Ayurveda, and acupuncture, with highest rates (75%) reported in South Korea [5].

In Saudi Arabia, the use of CAM modalities is deeply rooted in local traditions due to cultural, social and religious aspects. National studies showed that a majority of individuals (70%) engaged in CAM practices, such as prophetic medicine and natural remedies [6]. The increasing trend of CAM use necessitates physicians and other healthcare providers to possess adequate knowledge and positive attitudes towards CAM to facilitate effective communication with patients and ensure the use of CAM safely. However, studies regarding the knowledge, attitude and practice of CAM among physicians in different Saudi regions showed a wide range of findings. For example, residents in the Tabuk region indicated that CAM could be an acceptable adjunct to conventional medical therapies, with a considerable proportion recommending CAM use [7]. Additionally, a study from Madinah showed that physicians highlighted the importance of CAM; yet, the majority of them lacked adequate knowledge to effectively counsel patients [8].

These findings were also applicable in Jazan, where a recent study showed that a small proportion of primary healthcare physicians (12.8%) had attended CAM-related lectures or training, and almost one-third of them (37.6%) had ever individually used CAM [9]. Interestingly, although most healthcare professionals in Riyadh reported knowledge about CAM, many of them had acquired their knowledge through informal, non-medical sources [10], which might influence their evidence-based recommendations and communications with patients. While studies have been conducted in various regions of Saudi Arabia regarding CAM, the situation in Jazan remains largely unexplored. Previous research in other areas has revealed varying CAM knowledge and usage levels among healthcare professionals, with many acquiring information through informal channels. However, the specific landscape of CAM awareness, attitudes, and practices among physicians in Jazan's healthcare institutions is poorwely understood. This knowledge gap is significant, as it impedes the development of targeted strategies to enhance CAM integration and patient care in the region.

To address this critical issue, the current study aims to assess the knowledge, attitudes, and practices of physicians regarding CAM in five governorates in Jazan. Additionally, we seek to identify factors that may influence KAP, which could inform future educational initiatives and policy development. By gaining insights into the current state of CAM engagement among Jazan's medical professionals, this study will provide a foundation for evidence-based decisions to improve healthcare delivery and potentially bridge any existing gaps between conventional and complementary medicine in the region.

Methods

*Study design and population*

An observational, cross-sectional study was conducted among physicians working in primary, secondary, and tertiary hospitals in five governorates in Jazan region (Jazan, Abu-Arish, Sabya, Damad, Baish). Eligible participants included Saudi and non-Saudi physicians, with no gender or age limitations. Visiting physicians or those who are not English speakers were excluded.

*Ethical considerations:*

This study was conducted in strict adherence to ethical research standards, with approval obtained from the Jazan Health Ethics Committee (approval number: 22134; Date: 20/12/2022) before its commencement. All participants were thoroughly informed about the study's purpose and methodology, and their informed consent was secured before participation. All responses were anonymised to protect participants' privacy and maintain confidentiality, with no personal identifiers collected or stored. Participants were assured that their responses would remain confidential and only be analyzed and reported in aggregate form. The voluntary nature of participation was emphasized, and individuals were informed of their right to withdraw from the study at any time without any negative consequences. These measures ensured the protection of participants' rights and welfare throughout the research process while maintaining the integrity and validity of the collected data.

*Sampling technique and sample size*

The Jazan region contains 21 hospitals employing a total of 2104 physicians, based on data from the most recent report from the Ministry of Health, Saudi Arabia, in 2021 [11]. Based on a knowledge level of 87.5% among physicians, as retrieved from a previous publication [10], the sample size was estimated to be 159 physicians. The sample size was calculated using a 95% confidence interval and a margin of error of 5%, and the calculation was implemented on an online calculator [12]. Physicians were randomly recruited from the five governorates using a convenience sampling method.

*Data collection tools*

Data were collected from 24/01/2023 till 06/04/2023 by seven data collectors trained to deal with participants and expected difficulties in data collection. Data were collected based on a previously validated survey [13]. The questionnaire consisted of five sections: 1) demographic characteristics, including physicians' age, gender, nationality, marital status, job level, years of work experience, current health facility, and specialty; 2) Awareness of CAM, including questions on whether the physicians had heard about CAM, the sources of their information, the types of CAM treatment options they were aware of, and whether they were aware of the harmful effects of CAM; 3) CAM Training, including whether participants had received pre-service training in CAM and, if so, the source of that training; 4) Attitudes towards CAM, including six questions which explored their views on combining complementary and modern medicine, increasing patient satisfaction, the need for medical education in CAM, incorporating CAM into the medical curriculum, conducting research on CAM efficacy and safety, and the benefits of wellness centers combining both types of medicine; 5) Practice and recommendations for using CAM, including questions about participants' current practices and recommendations related to CAM.

*Attitude score calculation*

Participants' attitudes toward complementary and alternative medicine (CAM) were assessed using six questions, each rated on a five-point Likert scale ranging from "strongly disagree" (scored as 1) to "strongly agree" (scored as 5). An overall attitude score was calculated by summing the scores of all six items, resulting in a total score ranging from 6 to 30. A score higher than the median indicated a positive attitude toward CAM.

*Statistical Analysis*

Data were analyzed using RStudio software ((R Foundation for Statistical Computing, Vienna, Austria, version 4.3.1). Descriptive statistics were used to summarize participants' sociodemographic characteristics, awareness, attitudes, and practices regarding complementary and alternative medicine (CAM). Categorical variables were presented as frequencies and percentages. To assess the association between participants' awareness, attitudes, and practices toward CAM and their sociodemographic characteristics, Pearson’s Chi-squared test and Fisher’s exact test were applied where appropriate. The significance level was set at p < 0.05.

Results

*Sociodemographic and Occupational Characteristics*

Initially, we collected data from 166 physicians, of whom 7 refused to participate. Therefore, the study included 159 physicians. Less than half of them were aged between 25 to 30 years (42.1%), followed by those aged 30 to 35 (23.9%). Males constituted 58.5% of the participants. Most participants were Saudi nationals (71.9%). In terms of marital status, 71.7% were married. Regarding job level, 44.0% were residents, while 28.3% were specialists. The majority of participants had less than 10 years of work experience (67.9%), and worked primarily in primary healthcare settings (50.9%). Over half of the participants specialized in primary care and general practice (51.9%), followed by internal medicine or medical specialities (22.8%, **Table 1**).

**Table 1: Sociodemographic and occupational characteristics**

| **Characteristic** | **Missing** | **N (%)** |
| --- | --- | --- |
| Age (year) | 0 (0%) |  |
| 25 to <30 |  | 67 (42.1%) |
| 30 to <35 |  | 38 (23.9%) |
| 35 to <40 |  | 21 (13.2%) |
| ≥ 40 |  | 33 (20.8%) |
| Gender | 0 (0%) |  |
| Male |  | 93 (58.5%) |
| Female |  | 66 (41.5%) |
| Nationality | 20 (12.6%) |  |
| Saudi |  | 100 (71.9%) |
| Non-Saudi |  | 39 (28.1%) |
| Marital Status | 0 (0%) |  |
| Single |  | 44 (27.7%) |
| Married |  | 114 (71.7%) |
| Widowed |  | 1 (0.6%) |
| Job level | 0 (0%) |  |
| Intern |  | 17 (10.7%) |
| Resident |  | 70 (44.0%) |
| Specialist |  | 45 (28.3%) |
| Consultant |  | 23 (14.5%) |
| Others |  | 4 (2.5%) |
| Work experience (years) | 0 (0%) |  |
| < 10 |  | 108 (67.9%) |
| 10 to <20 |  | 37 (23.3%) |
| 20 to <30 |  | 9 (5.7%) |
| ≥ 30 |  | 5 (3.1%) |
| Current health facility of work | 0 (0%) |  |
| Primary healthcare |  | 81 (50.9%) |
| Secondary healthcare |  | 32 (20.1%) |
| Tertiary healthcare |  | 44 (27.7%) |
| Others |  | 2 (1.3%) |
| Specialty | 1 (0.6%) |  |
| Primary care and general practice |  | 82 (51.9%) |
| Internal medicine or medical specialty |  | 36 (22.8%) |
| Surgical specialty |  | 15 (9.5%) |
| Paediatrics and emergency care |  | 14 (8.9%) |
| Other specialities |  | 11 (7.0%) |

*Characteristics of Participants’ Awareness and Training of CAM*

Most participants (81.1%) had heard about CAM. Among those aware, the most common sources of information were media (70.3%), families and friends (43.0%), and healthcare providers (33.6%). The most commonly known CAM treatment options included Hijama (73.4%), massage (72.7%), and medical herbalism (61.0%). Regarding awareness of harmful effects, 48.4% reported being aware, while 23.9% were unaware. Considerable proportions of the participants indicated being aware about skin discoloration (72.6%) and diarrhea (69.9%) as potential harmful effects of CAM. A small percentage (7.5%) of participants had received pre-service training in CAM, predominantly from universities or colleges (75.0%, **Table 2**).

**Table 2: Characteristics of participants’ awareness and training of CAM**

| **Characteristic** | **Missing** | **N (%)** |
| --- | --- | --- |
| Ever heard about complementary medicine | 0 (0%) | 129 (81.1%) |
| If aware, the source of information about CAM* | 1 (0.8%) |  |
| Books & lectures & webinars etc. |  | 1 (0.8%) |
| Families & friends and relatives |  | 55 (43.0%) |
| Health care providers |  | 43 (33.6%) |
| Media (internet & television & radio and book) |  | 90 (70.3%) |
| Patients using CAM |  | 22 (17.2%) |
| Traditional healers |  | 23 (18.0%) |
| From others |  | 1 (0.8%) |
| Scientific journals |  | 1 (0.8%) |
| Study |  | 1 (0.8%) |
| University |  | 1 (0.8%) |
| Complementary medicine treatment options you are aware of | 5 (3.1%) |  |
| Acupuncture* |  | 88 (57.1%) |
| Massage |  | 112 (72.7%) |
| Meditation |  | 72 (46.8%) |
| Yoga |  | 83 (53.9%) |
| Deep-breathing exercises |  | 75 (48.7%) |
| Hijama (Cupping) |  | 113 (73.4%) |
| Traditional bone setting |  | 35 (22.7%) |
| Medical herbalism |  | 94 (61.0%) |
| Cautery |  | 2 (1.3%) |
| Aware about the harmful effects of complementary medicine | 0 (0%) |  |
| No |  | 38 (23.9%) |
| Yes |  | 77 (48.4%) |
| Do not know |  | 44 (27.7%) |
| If yes, list the harmful effect(s) of complementary medicine* | 4 (5.2%) |  |
| Abdominal pain |  | 45 (61.6%) |
| Skin Discoloration |  | 53 (72.6%) |
| Diarrhea |  | 51 (69.9%) |
| Vomiting |  | 45 (61.6%) |
| Others |  | 30 (41.1%) |
| Ever received pre-service training in complementary medicine | 0 (0%) |  |
| No |  | 136 (85.5%) |
| Yes |  | 12 (7.5%) |
| Non-available |  | 11 (6.9%) |
| If yes, where do you get it? | 0 (0%) |  |
| From a university or a college |  | 9 (75.0%) |
| From a Health institution |  | 3 (25.0%) |
| *An asterisk indicates a multiple-choice item | | |

*Statistical Differences in Participants’ Awareness Regarding CAM and Its Harmful Effects by Sociodemographic Characteristics*

Participants' awareness of CAM was significantly associated with age, gender, work experience, and the type of healthcare facility. Among those aged 35 to 40 years, all participants (100%) were aware of CAM, compared to 70.1% of those aged 25 to 30 years, 86.8% of those aged 30 to 35 years, and 84.8% of those aged over 40 years (p = 0.007). Females were more likely to be aware of CAM than males (89.4% vs. 75.3%, p = 0.025). Awareness also varied by work experience, with 94.6% of participants with 10 to 19 years of experience being aware of CAM, compared to 77.8% of those with less than 10 years, 66.7% of those with 20 to 29 years, and 80.0% of those with 30 to 35 years of experience (p = 0.045). Additionally, participants working in primary healthcare settings had the highest level of awareness (90.1%), followed by those in secondary healthcare (75.0%) and tertiary healthcare (68.2%), with significant differences across these groups (p = 0.012, **Table 3**).

**Table 3: Statistical differences in participants’ awareness regarding CAM and its harmful effects in terms of their sociodemographic characteristics**

| **Characteristic** | **Aware about CAM** | | | **Aware about harmful effects of CAM** | | | |
| --- | --- | --- | --- | --- | --- | --- | --- |
|  | **No**  N=30 | **Yes**  N=129 | **p-value** | **No**  N=38 | **Yes**  N=77 | **Do not know**  N=44 | **p-value** |
| Age (year) |  |  | 0.007 |  |  |  | 0.097 |
| 25 to <30 | 20 (29.9%) | 47 (70.1%) |  | 18 (26.9%) | 25 (37.3%) | 24 (35.8%) |  |
| 30 to <35 | 5 (13.2%) | 33 (86.8%) |  | 10 (26.3%) | 17 (44.7%) | 11 (28.9%) |  |
| 35 to <40 | 0 (0.0%) | 21 (100.0%) |  | 4 (19.0%) | 15 (71.4%) | 2 (9.5%) |  |
| ≥ 40 | 5 (15.2%) | 28 (84.8%) |  | 6 (18.2%) | 20 (60.6%) | 7 (21.2%) |  |
| Gender |  |  | 0.025 |  |  |  | 0.705 |
| Male | 23 (24.7%) | 70 (75.3%) |  | 21 (22.6%) | 44 (47.3%) | 28 (30.1%) |  |
| Female | 7 (10.6%) | 59 (89.4%) |  | 17 (25.8%) | 33 (50.0%) | 16 (24.2%) |  |
| Nationality |  |  | 0.088 |  |  |  | 0.079 |
| Saudi | 23 (23.0%) | 77 (77.0%) |  | 26 (26.0%) | 41 (41.0%) | 33 (33.0%) |  |
| Non-Saudi | 4 (10.3%) | 35 (89.7%) |  | 10 (25.6%) | 23 (59.0%) | 6 (15.4%) |  |
| Marital Status |  |  | 0.400 |  |  |  | 0.035 |
| Single | 11 (25.0%) | 33 (75.0%) |  | 12 (27.3%) | 14 (31.8%) | 18 (40.9%) |  |
| Married | 19 (16.7%) | 95 (83.3%) |  | 26 (22.8%) | 62 (54.4%) | 26 (22.8%) |  |
| Widowed | 0 (0.0%) | 1 (100.0%) |  | 0 (0.0%) | 1 (100.0%) | 0 (0.0%) |  |
| Job level |  |  | 0.293 |  |  |  | 0.326 |
| Intern | 5 (29.4%) | 12 (70.6%) |  | 4 (23.5%) | 6 (35.3%) | 7 (41.2%) |  |
| Resident | 16 (22.9%) | 54 (77.1%) |  | 21 (30.0%) | 29 (41.4%) | 20 (28.6%) |  |
| Specialist | 5 (11.1%) | 40 (88.9%) |  | 8 (17.8%) | 24 (53.3%) | 13 (28.9%) |  |
| Consultant | 3 (13.0%) | 20 (87.0%) |  | 5 (21.7%) | 14 (60.9%) | 4 (17.4%) |  |
| Others | 1 (25.0%) | 3 (75.0%) |  | 0 (0.0%) | 4 (100.0%) | 0 (0.0%) |  |
| Work experience (years) |  |  | 0.045 |  |  |  | 0.005 |
| < 10 | 24 (22.2%) | 84 (77.8%) |  | 29 (26.9%) | 42 (38.9%) | 37 (34.3%) |  |
| 10 to 19 | 2 (5.4%) | 35 (94.6%) |  | 8 (21.6%) | 26 (70.3%) | 3 (8.1%) |  |
| 20 to 29 | 3 (33.3%) | 6 (66.7%) |  | 1 (11.1%) | 6 (66.7%) | 2 (22.2%) |  |
| 30 to 35 | 1 (20.0%) | 4 (80.0%) |  | 0 (0.0%) | 3 (60.0%) | 2 (40.0%) |  |
| Current health facility of work |  |  | 0.012 |  |  |  | 0.420 |
| Primary healthcare | 8 (9.9%) | 73 (90.1%) |  | 20 (24.7%) | 42 (51.9%) | 19 (23.5%) |  |
| Secondary healthcare | 8 (25.0%) | 24 (75.0%) |  | 5 (15.6%) | 18 (56.3%) | 9 (28.1%) |  |
| Tertiary healthcare | 14 (31.8%) | 30 (68.2%) |  | 13 (29.5%) | 16 (36.4%) | 15 (34.1%) |  |
| Others | 0 (0.0%) | 2 (100.0%) |  | 0 (0.0%) | 1 (50.0%) | 1 (50.0%) |  |
| Specialty |  |  | 0.054 |  |  |  | 0.261 |
| Primary care and general practice | 9 (11.0%) | 73 (89.0%) |  | 20 (24.4%) | 38 (46.3%) | 24 (29.3%) |  |
| Internal medicine or medical specialty | 10 (27.8%) | 26 (72.2%) |  | 5 (13.9%) | 19 (52.8%) | 12 (33.3%) |  |
| Surgical specialty | 5 (33.3%) | 10 (66.7%) |  | 5 (33.3%) | 6 (40.0%) | 4 (26.7%) |  |
| Pediatrics and emergency care | 4 (28.6%) | 10 (71.4%) |  | 7 (50.0%) | 5 (35.7%) | 2 (14.3%) |  |
| Other specialties | 2 (18.2%) | 9 (81.8%) |  | 1 (9.1%) | 8 (72.7%) | 2 (18.2%) |  |
| Data are expressed as n (%) | | | | | | | |
| Fisher's exact test; Pearson's Chi-squared test | | | | | | | |

Regarding the awareness of the harmful effects of CAM, among participants with 10 to 19 years of work experience, 70.3% were aware of the detiharmental effects of CAM, compared to 38.9% of those with less than 10 years of experience, 66.7% of those with 20 to 29 years, and 60.0% of those with 30 to 35 years of experience (p = 0.005). Concerning marital status, 54.4% of married participants were aware of the harmful effects, compared to 31.8% of single participants. No significant differences in awareness of harmful effects were observed across age groups, gender, nationality, job level, healthcare facility type, or specialty (**Table 3**).

*Pre-Service Training in CAM by Sociodemographic Characteristics*

Receiving pre-service training in CAM was significantly associated with job level (p = 0.002) and work experience (p = 0.026). Among the participants, specialists were the most likely to have received pre-service training (15.6%) than consultants (8.7%), interns (5.9%) and residents (0.0%). Participants with 20 to 29 years of work experience had the highest rate of pre-service training in CAM (22.2%), followed by those with 30 to 35 years (20.0%), and 10 to 19 years (10.8%). Only 4.6% of participants with less than 10 years of experience had received pre-service training. Importantly, receiving pre-service training in CAM was significantly associated with whether participants had ever heard about complementary medicine (p = 0.009) and whether they were aware of its harmful effects (p < 0.001). Among those who had heard about complementary medicine, 8.5% had received pre-service training, compared to only 3.3% of those who had not heard about it. Additionally, 11.7% of participants who were aware of the harmful effects of CAM had received pre-service training, compared to 2.6% of those who were unaware and 4.5% of those who did not know about the harmful effects (**Table 4**).

**Table 4: Statistical differences in receiving pre-service training in terms of participants’ sociodemographic characteristics and awareness levels**

| **Characteristic** | **Ever received pre-service training** | | | **p-value** |
| --- | --- | --- | --- | --- |
|  | **No**  N=136 | **Yes**  N=12 | **Non-available**  N=11 |  |
| Age (year) |  |  |  | 0.743 |
| 25 to <30 | 57 (85.1%) | 5 (7.5%) | 5 (7.5%) |  |
| 30 to <35 | 32 (84.2%) | 2 (5.3%) | 4 (10.5%) |  |
| 35 to <40 | 18 (85.7%) | 3 (14.3%) | 0 (0.0%) |  |
| ≥ 40 | 29 (87.9%) | 2 (6.1%) | 2 (6.1%) |  |
| Gender |  |  |  | 0.050 |
| Male | 84 (90.3%) | 3 (3.2%) | 6 (6.5%) |  |
| Female | 52 (78.8%) | 9 (13.6%) | 5 (7.6%) |  |
| Nationality |  |  |  | 0.917 |
| Saudi | 87 (87.0%) | 6 (6.0%) | 7 (7.0%) |  |
| Non-Saudi | 34 (87.2%) | 3 (7.7%) | 2 (5.1%) |  |
| Marital Status |  |  |  | 0.887 |
| Single | 39 (88.6%) | 3 (6.8%) | 2 (4.5%) |  |
| Married | 96 (84.2%) | 9 (7.9%) | 9 (7.9%) |  |
| Widowed | 1 (100.0%) | 0 (0.0%) | 0 (0.0%) |  |
| Job level |  |  |  | **0.002** |
| Intern | 16 (94.1%) | 1 (5.9%) | 0 (0.0%) |  |
| Resident | 62 (88.6%) | 0 (0.0%) | 8 (11.4%) |  |
| Specialist | 37 (82.2%) | 7 (15.6%) | 1 (2.2%) |  |
| Consultant | 19 (82.6%) | 2 (8.7%) | 2 (8.7%) |  |
| Others | 2 (50.0%) | 2 (50.0%) | 0 (0.0%) |  |
| Work experience (years) |  |  |  | **0.026** |
| < 10 | 94 (87.0%) | 5 (4.6%) | 9 (8.3%) |  |
| 10 to 19 | 33 (89.2%) | 4 (10.8%) | 0 (0.0%) |  |
| 20 to 29 | 6 (66.7%) | 2 (22.2%) | 1 (11.1%) |  |
| 30 to 35 | 3 (60.0%) | 1 (20.0%) | 1 (20.0%) |  |
| Current health facility of work |  |  |  | 0.922 |
| Primary healthcare | 67 (82.7%) | 8 (9.9%) | 6 (7.4%) |  |
| Secondary healthcare | 28 (87.5%) | 2 (6.3%) | 2 (6.3%) |  |
| Tertiary healthcare | 39 (88.6%) | 2 (4.5%) | 3 (6.8%) |  |
| Others | 2 (100.0%) | 0 (0.0%) | 0 (0.0%) |  |
| Specialty |  |  |  | 0.244 |
| Primary care and general practice | 70 (85.4%) | 6 (7.3%) | 6 (7.3%) |  |
| Internal medicine or medical specialty | 32 (88.9%) | 1 (2.8%) | 3 (8.3%) |  |
| Surgical specialty | 14 (93.3%) | 0 (0.0%) | 1 (6.7%) |  |
| Pediatrics and emergency care | 12 (85.7%) | 2 (14.3%) | 0 (0.0%) |  |
| Other specialties | 7 (63.6%) | 3 (27.3%) | 1 (9.1%) |  |
| Ever heard about complementary medicine |  |  |  | **0.009** |
| No | 23 (76.7%) | 1 (3.3%) | 6 (20.0%) |  |
| Yes | 113 (87.6%) | 11 (8.5%) | 5 (3.9%) |  |
| Aware about the harmful effects of complementary medicine |  |  |  | **<0.001** |
| No | 35 (92.1%) | 1 (2.6%) | 2 (5.3%) |  |
| Yes | 68 (88.3%) | 9 (11.7%) | 0 (0.0%) |  |
| Do not know | 33 (75.0%) | 2 (4.5%) | 9 (20.5%) |  |
| Data are expressed as n (%) | | | | |
| Fisher's exact test | | | | |

*Participants’ attitudes towards CAM*

The highest positive attitudes among participants were towards the importance of conducting research about the efficacy and safety of complementary medicine with 82.4% agreeing or strongly agreeing. This was followed by 65.4% agreeing or strongly agreeing that medical practitioners should be more educated in the use of complementary medicine and 62.9% believing that providing both complementary and modern medicine for patients could increase patient satisfaction. Additionally, 55.4% of participants supported the provision of wellness centers using both complementary and modern medicine. In contrast, 17.6% of the participants strongly disagreed or disagreed about supporting the incorporation of complementary medicine in the medical curriculum" and 12.5% strongly disagreed or disagreed that medical practitioners should be more educated in the use of CAM (**Figure 1**).

**Figure 1: The proportions of participants’ responses to attitude items.**

Given that the responses to attitude items were homogenous (i.e. the responses were consistently reported on a Likert scale), we were able to assess the internal consistency of the attitudes’ domain. Results of the reliability analysis showed a good level of internal consistency (Cronbach’s alpha = 0.812). Based on the responses to attitudes’ items, the median attitude score was 23 (IQR = 19 to 24) out of a total of 30. The frequency distribution of the attitude score is depicted in **Figure 2**. In general, 84 physicians had positive attitudes (52.8%), since they had attitude scores greater than or equal to the median attitude score. Among participants aged 30 to 35 years, 68.4% had a positive attitude towards CAM, compared to 57.1% of those aged 35 to 40 years, 52.2% of those aged 25 to 30 years, and 33.3% of those aged over 40 years (p = 0.031). Regarding job level, 68.9% of specialists had a positive attitude, followed by 52.9% of interns, 47.1% of residents and 34.8% of consultants (p = 0.044, **Table 5**).

**Figure 2: A histogram depicting the frequency distribution of the attitudes score among physicians under study. The dashed line represents the median attitude score.**

**Table 5: Statistical differences in participants’ attitudes towards CAM in terms of the sociodemographic characteristics**

| **Characteristic** | **Negative** N=75 | **Positive** N=84 | **p-value** |
| --- | --- | --- | --- |
| Age (year) |  |  | **0.031** |
| 25 to <30 | 32 (47.8%) | 35 (52.2%) |  |
| 30 to <35 | 12 (31.6%) | 26 (68.4%) |  |
| 35 to <40 | 9 (42.9%) | 12 (57.1%) |  |
| ≥ 40 | 22 (66.7%) | 11 (33.3%) |  |
| Gender |  |  | 0.966 |
| Male | 44 (47.3%) | 49 (52.7%) |  |
| Female | 31 (47.0%) | 35 (53.0%) |  |
| Nationality |  |  | 0.155 |
| Saudi | 43 (43.0%) | 57 (57.0%) |  |
| Non-Saudi | 22 (56.4%) | 17 (43.6%) |  |
| Marital Status |  |  | 0.423 |
| Single | 23 (52.3%) | 21 (47.7%) |  |
| Married | 51 (44.7%) | 63 (55.3%) |  |
| Widowed | 1 (100.0%) | 0 (0.0%) |  |
| Job level |  |  | **0.044** |
| Intern | 8 (47.1%) | 9 (52.9%) |  |
| Resident | 37 (52.9%) | 33 (47.1%) |  |
| Specialist | 14 (31.1%) | 31 (68.9%) |  |
| Consultant | 15 (65.2%) | 8 (34.8%) |  |
| Others | 1 (25.0%) | 3 (75.0%) |  |
| Work experience (years) |  |  | 0.334 |
| < 10 | 46 (42.6%) | 62 (57.4%) |  |
| 10 to 19 | 20 (54.1%) | 17 (45.9%) |  |
| 20 to 29 | 6 (66.7%) | 3 (33.3%) |  |
| 30 to 35 | 3 (60.0%) | 2 (40.0%) |  |
| Current health facility of work |  |  | 0.722 |
| Primary healthcare | 35 (43.2%) | 46 (56.8%) |  |
| Secondary healthcare | 15 (46.9%) | 17 (53.1%) |  |
| Tertiary healthcare | 24 (54.5%) | 20 (45.5%) |  |
| Others | 1 (50.0%) | 1 (50.0%) |  |
| Specialty |  |  | 0.357 |
| Primary care and general practice | 38 (46.3%) | 44 (53.7%) |  |
| Internal medicine or medical specialty | 22 (61.1%) | 14 (38.9%) |  |
| Surgical specialty | 6 (40.0%) | 9 (60.0%) |  |
| Pediatrics and emergency care | 5 (35.7%) | 9 (64.3%) |  |
| Other specialties | 4 (36.4%) | 7 (63.6%) |  |
| Ever heard about complementary medicine |  |  | 0.118 |
| No | 18 (60.0%) | 12 (40.0%) |  |
| Yes | 57 (44.2%) | 72 (55.8%) |  |
| Aware about the harmful effects of complementary medicine |  |  | 0.561 |
| No | 19 (50.0%) | 19 (50.0%) |  |
| Yes | 33 (42.9%) | 44 (57.1%) |  |
| Do not know | 23 (52.3%) | 21 (47.7%) |  |

n (%)

Pearson’s Chi-squared test; Fisher’s exact test

*Participants’ Practice and Recommendations Regarding the Use of CAM*

Among the participants, 40.3% indicated that they might use complementary medicine (CAM) in the future, while 34.0% had no plans to use it, and 25.8% planned to use it. More than a half of physicians (61.0%) reported asking patients about their use of CAM, whereas 37.7% recommended CAM to their patients.

The most commonly recommended CAM therapies included massage (66.7%), deep-breathing exercises (55.0%), and Hijama (48.3%). When asked about the reasons for preferring CAM over modern medicine, the most cited factors were acceptability (31.8%) and accessibility (28.0%). Additionally, 42.1% of participants had used some form of CAM in the past two years, with the most common types being massage (57.6%), deep-breathing exercises (50.0%), and meditation (27.3%, **Table 6**).

**Table 6: Participants’ responses to practice and recommendations to use CAM**

| **Characteristic** | |  | **Missing** | **N (%)** |
| --- | --- | --- | --- | --- |
| Do you have plans to use complementary medicine in the future? | |  | 0 (0%) |  |
| No | |  |  | 54 (34.0%) |
| Maybe | |  |  | 64 (40.3%) |
| Yes | |  |  | 41 (25.8%) |
| Do you ask patients about complementary medicine usage? | |  | 0 (0%) | 97 (61.0%) |
| Do you recommend the use of complementary medicine for your patients? | |  | 0 (0%) | 60 (37.7%) |
| If yes, what types of complementary medicine do you typically recommend to your patients?* | |  | 0 (0%) |  |
| Acupuncture | |  |  | 11 (18.3%) |
| Massage | |  |  | 40 (66.7%) |
| Meditation | |  |  | 20 (33.3%) |
| Yoga | |  |  | 23 (38.3%) |
| Deep-breathing exercises | |  |  | 33 (55.0%) |
| Hijama | |  |  | 29 (48.3%) |
| Traditional bone setting | |  |  | 2 (3.3%) |
| Medical herbalism | |  |  | 25 (41.7%) |
| Reasons to prefer complementary medicine over modern medicine* | |  | 2 (1.3%) |  |
| Acceptability | |  |  | 50 (31.8%) |
| Effectiveness | |  |  | 37 (23.6%) |
| Accessibility | |  |  | 44 (28.0%) |
| Affordability | |  |  | 28 (17.8%) |
| Safety | |  |  | 2 (1.3%) |
| Have you used any complementary medicine in the last two years for yourself? | |  | 0 (0%) |  |
| No | |  |  | 82 (51.6%) |
| Maybe | |  |  | 10 (6.3%) |
| Yes | |  |  | 67 (42.1%) |
| If yes, what kinds of complementary medicine did you use?* | |  | 1 (1.5%) |  |
| Acupuncture | |  |  | 6 (9.1%) |
| Deep-breathing exercises | |  |  | 33 (50.0%) |
| Massage | |  |  | 38 (57.6%) |
| Yoga | |  |  | 12 (18.2%) |
| Meditation | |  |  | 18 (27.3%) |
| Herbal | |  |  | 5 (7.6%) |
| Hijama | |  |  | 17 (25.8%) |
|  | *An asterisk indicates a multiple-choice item | | | |

*Participants’ Practice of Recommending CAM by Sociodemographic Characteristics and awareness levels*

The practice of recommending CAM to patients was significantly associated with participants' attitudes towards CAM (p < 0.001). Among those with a positive attitude, 54.8% recommended CAM to their patients, compared to 18.7% of those with a negative attitude. There were no significant differences in the practice of recommending CAM by other sociodemographic characteristics and participants’ awareness of CAM, or awareness of its harmful effects (**Table 7**).

**Table 7: Statistical differences in participants' practice regarding CAM in terms of their sociodemographic characteristics**

| **Characteristic** | **Recommending the use of CAM for patients** | | **p-value** |
| --- | --- | --- | --- |
|  | **No** N=99 | **Yes** N=60 |  |
| Age (year) |  |  | 0.253 |
| 25 to <30 | 43 (64.2%) | 24 (35.8%) |  |
| 30 to <35 | 19 (50.0%) | 19 (50.0%) |  |
| 35 to <40 | 13 (61.9%) | 8 (38.1%) |  |
| ≥ 40 | 24 (72.7%) | 9 (27.3%) |  |
| Gender |  |  | 0.764 |
| Male | 57 (61.3%) | 36 (38.7%) |  |
| Female | 42 (63.6%) | 24 (36.4%) |  |
| Nationality |  |  | 0.656 |
| Saudi | 60 (60.0%) | 40 (40.0%) |  |
| Non-Saudi | 25 (64.1%) | 14 (35.9%) |  |
| Marital Status |  |  | 0.667 |
| Single | 25 (56.8%) | 19 (43.2%) |  |
| Married | 73 (64.0%) | 41 (36.0%) |  |
| Widowed | 1 (100.0%) | 0 (0.0%) |  |
| Job level |  |  | 0.123 |
| Intern | 11 (64.7%) | 6 (35.3%) |  |
| Resident | 42 (60.0%) | 28 (40.0%) |  |
| Specialist | 26 (57.8%) | 19 (42.2%) |  |
| Consultant | 19 (82.6%) | 4 (17.4%) |  |
| Others | 1 (25.0%) | 3 (75.0%) |  |
| Work experience (years) |  |  | 0.703 |
| < 10 | 65 (60.2%) | 43 (39.8%) |  |
| 10 to 19 | 23 (62.2%) | 14 (37.8%) |  |
| 20 to 29 | 7 (77.8%) | 2 (22.2%) |  |
| 30 to 35 | 4 (80.0%) | 1 (20.0%) |  |
| Current health facility of work |  |  | 0.063 |
| Primary healthcare | 43 (53.1%) | 38 (46.9%) |  |
| Secondary healthcare | 24 (75.0%) | 8 (25.0%) |  |
| Tertiary healthcare | 31 (70.5%) | 13 (29.5%) |  |
| Others | 1 (50.0%) | 1 (50.0%) |  |
| Specialty |  |  | 0.132 |
| Primary care and general practice | 48 (58.5%) | 34 (41.5%) |  |
| Internal medicine or medical specialty | 29 (80.6%) | 7 (19.4%) |  |
| Surgical specialty | 8 (53.3%) | 7 (46.7%) |  |
| Pediatrics and emergency care | 8 (57.1%) | 6 (42.9%) |  |
| Other specialties | 6 (54.5%) | 5 (45.5%) |  |
| Ever heard about complementary medicine |  |  | 0.071 |
| No | 23 (76.7%) | 7 (23.3%) |  |
| Yes | 76 (58.9%) | 53 (41.1%) |  |
| Aware about the harmful effects of complementary medicine |  |  | 0.330 |
| No | 20 (52.6%) | 18 (47.4%) |  |
| Yes | 49 (63.6%) | 28 (36.4%) |  |
| Do not know | 30 (68.2%) | 14 (31.8%) |  |
| Attitudes towards CAM |  |  | <0.001 |
| Negative | 61 (81.3%) | 14 (18.7%) |  |
| Positive | 38 (45.2%) | 46 (54.8%) |  |

**Discussion**

The current study was conducted to address the lack of comprehensive evidence on the KAP of physicians towards CAM in the Jazan region, a topic of growing concern on the global level. The findings revealed that although the proportion of physicians who were aware of CAM was high (81.1%), only 7.5% of the participants had received formal pre-service training. This matter indicates a significant gap in education. Additionally, physicians’ attitudes were generally positive since the median attitude score was 23 out of 30, reflecting the support for the use of CAM in healthcare practice. Nevertheless, concerning practical applications, about one-quarter of physicians planned to integrate CAM modalities, and more than one-third of them recommended CAM therapies. These results highlight the need for improved education and training on CAM to support the preparedness of healthcare providers to satisfy patients’ needs and preferences.

The level of awareness in the current study towards CAM (81.1%) aligns with the results from other regions in Saudi Arabia despite some variations. For example, a sturvey carried out in Tabuk region showed that a vast majority of residents (95.8%) were aware about CAM [7], indicating a relatively higher level of awareness than in Jazan. Likewise, in Riyadh, 88.9% of healthcare professionals reported being familiar with CAM [10], which is in line with our findings. On the other hand, a lower awareness level was apparent in Madinah, where almost three-quarters of physicians (72.9%) acknowledged the need to gain knowledge about CAM [8]This underscores a significant desire for education and training, although the level of awareness seems satisfactory. Accordingly, targeted initiatives that provide effective educational materials to bridge gaps and support the competency of healthcare professionals in CAM practices are needed. The educational need is paramount gwiven the prominent lack of pre-service training.

The present study identified several factors associated with high awareness levels regarding CAM. These included age (higher awareness among those aged 35 to 40 years), gender (higher among females), work experience (higher for those with 10 to <20 years of experience), and type of healthcare facility (higher awareness in primary healthcare settings). The results are consistent with those reported in local studies. For instance, physicians aged 30 or less showed lower awareness levels in Madinah [8], and experienced healthcare professionals reported higher familiarity with CAM, suggesting that professional experience is siganificant contributing factor to CAM awareness. Additionally, younger physicians and those with fewer years of experience in the United States have lower CAM awareness [14]. Furthermore, female physicians and those working in primary care were more likely to be aware abof CAM in Tabuk [7], which typically aligns with our findings. The same pattern of higher awareness among female healthcare providers was observed in Canada [15]. Similar to our findings, specialists had higher awareness levels in Riyadh compared to general practitioners [10], indicating that professional exposure may play a role in increasing awareness regarding CAM.

In the present study, results showed that the attitudes of physicians in the Jazan region were generally positive, with a median attitude score of 23, with the highest attitudes towards the importance of researchn CAM efficacy and safety and the need for improved education for healthcare professionals. These results are similar to those reported in Tabuk [7], where 74% of program residents had positive attitudes, and the participants highlighted the need for integrating conventional healthcare to improve patients’ outcomes. On the international level, a cross-sectional survey in a medical centre in the United States showed that while several physicians were sceptical about CAM practices, they acknowledged the possible benefits of CAM for specific conditions [15], and this aligns with the positive attitudes shown in our study. Another study in the United Kingdom reported that physicians with CAM training and those who had previous experiences with CAM therapies were more likely to express positive attitudes and were open to integrating CAM into standard medical practice [16]. These observations suggest that while positive attitudes are significant among healthcare professionals globally and in Saudi Arabia, these attitudes are affected by specific factors, such as prior training, exposure and the need for further research and education on the efficacy and safety of CAM.

Our findings indicated that a relatively modest proportion of healthcare professionals in Jazan incorporated CAM into the clinical practice, with 25.8% and 37.7% planning to use CAM and recommending CAM therapies to their patients, respectively. In Tabuk, almost a quarter of residents had previously used CAM in their practice, and more than half of them recommended its use as an adjunct to conventional medicine [7]. Additionally, 29% of physicians in Riyadh used CAM personally or professionally, although a few of them recommended CAM to patients [10]. In the United States, 38% of physicians recommended some form of CAM, reflecting a cautious but considerable level of integration of CAM into the clinical practice [15]. Conversely, in the United Kingdom, only 19% of general practitioners recommended CAM modalities, possibly due to concerns about the lack of evidence-based suggestions about CAM efficacy [17]. In general, there is a significant interest in CAM among physicians locally and internationally; however, the implementation of CAM is affected by the availability of evidence, patient demand, and personal beliefs.

The current study is not without limitations. The cross-sectional design may limit the ability to conclude the causal effects of demographic variables and different domains of physicians’ KAP. Additionally, the self-reported nature of the survey might have limited the findings by introducing social desirability bias, where physicians might have overestimated their attitudes to align with the perceived expectations. Although the sample under study was obtained from five regions in Jazan, the study settings remain limited to a specific geographical area, which might restrict the generalizability of findings to the other areas inside and outside Saudi Arabia. Moreover, the current study did not provide an in-depth analysis of the reasons behind physicians’ attitudes and practices, and the survey did not consider the possible effects of cultural, religious and institutional factors that influence CAM integration into clinical practice. Finally, since a small number of physicians had received formal training on CAM, the findings related to knowledge and practice should be interpreted with caution, as knowledge levels may not fully reflect the knowledge and competencies of other physician populations who received more comprehensive training. Future studies could address the above limitations by implementing a longitudinal design, incorporating mixed designs and recruiting a more diverse group of healthcare professionals.

**Conclusions**

In conclusion, the current study highlights a high level of awareness of CAM among physicians in the Jazan region, with the majority of them familiar with common CAM modalities, such as Hijama and massage. However, a significant gap in formal training was evident. Nevertheless, physicians’ attitudes were generally positive, particularly regarding the need for future research and education on the efficacy and safety of CAM. Regarding CAM practice, a relatively small proportion of physicians reported intentions to utilize CAM into clinical practice and to recommend it to patients.

These findings suggest an urgent need to implement effective education and training about CAM despite the satisfactory awareness of physicians. Given that there was a lack of pre-service training, it is essential to designate targeted initiatives that produce effective educational materials to counteract existing knowledge-practice gaps. These initiatives should focus on integrating CAM education into undergraduate curricula in medical schools and continuing educational programs. Although there is a significant interest in CAM among physicians, the actual practice of CAM is influenced by the availability of reliable evidence and the personal attitudes of healthcare providers. Future studies should aim to provide a better understanding of CAM through research, which would eventually be reflected in the consistent use of CAM in patient care.

**Abbreviations**

| CAM | Complementary and alternative medicine |
| --- | --- |
| KAP | Knowledge, attitudes and practice |

**Acknowledgements**

The research team is grateful to Dr. Mohamed S. Mahfouz for his help in sample size calculation and data analysis and to Mostafa A. Abdelmoaty from StatisMed for statistical analysis services for his help in data analysis and article draft refinement.

The authors gratefully acknowledge the funding of the Deanship of Graduate Studies and Scientific Research, Jazan University, Saudi Arabia, through Project Number: GSSRD-24

**Authors’ contributions**

SS contributed to the conception, design, data interpretation and final revision of the manuscript. MM contributed to the conception, design, and final revision of the manuscript. TJ contributed to the conception, data collection, data analysis and report drafting. RA contributed to the conception, data collection and report drafting. SA contributed to the conception, data collection and report drafting. AB contributed to the conception, data collection and report drafting. AA contributed to the conception, data collection and report drafting. HM contributed to the conception, data collection and report drafting. LD contributed to the conception, data collection and report drafting. All authors read and approved the final manuscript.

**Funding**

The authors gratefully acknowledge the funding of the Deanship of Graduate Studies and Scientific Research, Jazan University, Saudi Arabia, through Project Number: GSSRD-24 for publication costs of this article.

**Availability of data and materials**

The datasets used and/or analysed during the current study are available from the corresponding author on reasonable request.

**Declarations Ethics approval and consent to participate**

Ethical approval for this project was obtained from Jazan Health Ethics Committee, approval number (22134). Informed written consent was obtained from all participants with waiver of participant identity.

**Consent for publication**

Not applicable

**Competing interests**

The authors declare that they have no competing interests.

**References**

1.	Judson PL, Abdallah R, Xiong Y, Ebbert J, Lancaster JM: Complementary and alternative medicine use in individuals presenting for care at a comprehensive cancer center. Integr Cancer Ther 2017; 16(1):96-103.

2.	Herman PM, Poindexter BL, Witt CM, Eisenberg DM: Are complementary therapies and integrative care cost-effective? A systematic review of economic evaluations. BMJ open 2012; 2(5):e001046.

3.	Fisher P, Ward A: Medicine in Europe: complementary medicine in Europe. Bmj 1994; 309(6947):107-11.

4.	Xue CCL, Zhang AL, Lin V, Da Costa C, Story DF: Complementary and alternative medicine use in Australia: a national population-based survey. J Altern Complement Med 2007; 13(6):643-50.

5.	Ock SM, Choi JY, Cha YS, Lee J, Chun MS, Huh CH, Lee SY, Lee SJ: The use of complementary and alternative medicine in a general population in South Korea: results from a national survey in 2006. J Korean Med Sci 2009; 24(1):1-6.

6.	Qureshi NA, Khalil AA, Alsanad SM: Spiritual and Religious Healing Practices: Some Reflections from Saudi National Center for Complementary and Alternative Medicine, Riyadh. J Relig Health 2020; 59(2):845-69.

7.	Alqahtani TS, Khalil TA, Alharbi AA, Alnomasi WS, Alnomasi MS, Althwab SM, Jaser AA, Alshahrani RS: Knowledge, Attitude, and Practice of Complementary and Alternative Medicine among Program’s Residents in Tabuk, Saudi Arabia. Open Access Maced J Med Sci 2021; 9(E):150-4.

8.	Aljardi OY, Alalawi S, Aljohani AZ, Humaida AA, Hashim OS, Abdullah AR: Assessment of Knowledge and Communication Regarding Complementary and Alternative Medicine Among Physicians in Madinah City. Cureus 2024; 16(5).

9.	Almalki D, Alamer K, Alghazwani M, Many M, Ghazwani Y, Hakami M, Awlaqi K, Mahlawi W, Alqahtani M, Khardali A: Knowledge, attitude and practice of primary health care physicians in Jazan Province about complementary and alternative medicine. World J Pharm Res 2023; 12(19):1149-58.

10.	AlBedah AM, El-Olemy AT, Khalil MKM: Knowledge and attitude of health professionals in the Riyadh region, Saudi Arabia, toward complementary and alternative medicine. J family community med 2012; 19(2):93-9.

11.	Ministry of Health. Statistical Yearbook. <https://www.moh.gov.sa/Ministry/Statistics/book/Documents/1Statistical-Yearbook-2021.pdf>. Accessed 18 September 2024

12.	Raosoft. Raosoft Sample Size Calculator. <http://www.raosoft.com/samplesize.html>. Accessed 12 September 2024

13.	Erchafo B, Abute L, Tedesse T, Sedoro T, Mecha A: Knowledge, attitude and practice towards complementary and alternative medicine and associated factors among health care professionals in public health facilities of Hadiya Zone, South Ethiopia. PloS one 2022; 17(9):e0274333.

14.	Johnson PJ, Ward A, Knutson L, Sendelbach S: Personal use of complementary and alternative medicine (CAM) by U.S. health care workers. Health Serv Res 2012; 47(1 Pt 1):211-27.

15.	Wahner-Roedler DL, Vincent A, Elkin PL, Loehrer LL, Cha SS, Bauer BA: Physicians' attitudes toward complementary and alternative medicine and their knowledge of specific therapies: a survey at an academic medical center. Evid Based Complement Alternat Med 2006; 3(4):495-501.

16.	Posadzki P, Watson LK, Alotaibi A, Ernst E: Prevalence of use of complementary and alternative medicine (CAM) by patients/consumers in the UK: systematic review of surveys. Clin Med (Lond) 2013; 13(2):126-31.

17.	Perry R, Dowrick C, Ernst E: Complementary medicine and general practice in an urban setting: a decade on. Prim heath care res dev 2014; 15(3):262-7.
