# Supplementary material for: Bridging the knowledge-practice gap: A cross-sectional survey assessing physician knowledge, attitude and practice toward complementary and alternative medicine
Source: PLoS One. 2025 May 14;20(5):e0322613. doi: 10.1371/journal.pone.0322613 (PMC12077789; doi:10.1371/journal.pone.0322613)
Supplement: Attachment — Submitted filename: Comments PLOS ONE.docx [file pone.0322613.s005.docx]

Dear Editor, the authors need to improve the following;

1. The title should be changed by adding “Alternative “ on the phrase complementary medicine i.e CAM
2. The abstract needs to be written according to the journal guidelines. The statistics reported in the results section need improvement, e.g.; female physicians were not mentioned anywhere.
3. Authors must provide references for most of the statements used in the manuscript.
4. Authors should not generalise their findings to health practitioners while they only focus on physicians, as it can lead to misleading conclusions.
5. A sample size calculation formula should be indicated, as it ensures the study is statistically valid and reliable.
6. The results section needs to be summarised. Not everything should be presented, especially tables.
7. The discussion section and the conclusion section have to be improved.
8. More comments and suggestions have been indicated in the manuscript as track changes.
