# Supplementary material for: Bridging the knowledge-practice gap: A cross-sectional survey assessing physician knowledge, attitude and practice toward complementary and alternative medicine
Source: PLoS One. 2025 May 14;20(5):e0322613. doi: 10.1371/journal.pone.0322613 (PMC12077789; doi:10.1371/journal.pone.0322613)
Supplement: Attachment — Submitted filename: Response to reviewers.docx [file pone.0322613.s006.docx]

PONE-D-24-46538

Bridging the Knowledge-Practice Gap: A Cross-sectional Survey Assessing Physician Knowledge, Attitude and Practice toward Complementary Medicine

Dear respected editor and reviewers,

Thank you very much for allowing us to revise our manuscript. We are grateful to the Editor and reviewers for evaluating our manuscript and providing constructive comments to improve its quality. We have revised the manuscript to address all the points raised during the review process. Kindly find our responses to the outstanding reviewers in the following table:

**Journal Comments**

| Journal Requirements | Responses |
| --- | --- |
| 1. Please ensure that your manuscript meets PLOS ONE's style requirements, including those for file naming. The PLOS ONE style templates can be found at  https://journals.plos.org/plosone/s/file?id=wjVg/PLOSOne_formatting_sample_main_body.pdf and  https://journals.plos.org/plosone/s/file?id=ba62/PLOSOne_formatting_sample_title_authors_affiliations.pdf | The manuscript was amended according to the PLOS ONE style except for the results and discussion section. The results section is extensive in this manuscript and the authors felt that combining it with the discussion would make things confusing for readers. However, we are willing to separate these two sections if the journal requires so. We appreciate your understanding. |
| 2. Thank you for stating the following financial disclosure:   The authors gratefully acknowledge the funding of the Deanship of Graduate Studies and Scientific Research, Jazan University, Saudi Arabia, through Project Number: GSSRD-24 against publication costs of this article.  Please state what role the funders took in the study.  If the funders had no role, please state: ""The funders had no role in study design, data collection and analysis, decision to publish, or preparation of the manuscript.""  If this statement is not correct you must amend it as needed.  Please include this amended Role of Funder statement in your cover letter; we will change the online submission form on your behalf. | Financial disclosure was amended as follow: The authors gratefully acknowledge the funding of the Deanship of Graduate Studies and Scientific Research, Jazan University, Saudi Arabia, through Project Number: GSSRD-24. The funders had no role in study design, data collection and analysis, decision to publish, or preparation of the manuscript and support was for publication costs only. |
| 3. Thank you for stating the following in the Acknowledgments Section of your manuscript:  The research team is grateful to the help and support of Dr. Mohamed S. Mahfouz in sample size calculation and data analysis. The team is also grateful to the help and support of Mostafa A. Abdelmoaty from StatisMed for statistical analysis services for his help in data analysis and article draft refinement. The authors gratefully acknowledge the funding of the Deanship of Graduate Studies and Scientific Research, Jazan University, Saudi Arabia, through Project Number: GSSRD-24  We note that you have provided funding information that is not currently declared in your Funding Statement. However, funding information should not appear in the Acknowledgments section or other areas of your manuscript. We will only publish funding information present in the Funding Statement section of the online submission form.  Please remove any funding-related text from the manuscript and let us know how you would like to update your Funding Statement. Currently, your Funding Statement reads as follows:   The authors gratefully acknowledge the funding of the Deanship of Graduate Studies and Scientific Research, Jazan University, Saudi Arabia, through Project Number: GSSRD-24 against publication costs of this article.  Please include your amended statements within your cover letter; we will change the online submission form on your behalf. | We removed any funding-related text from the manuscript and updated the funding statement as mentioned above. |
| 4. Ethics statement appears in the Methods section of the manuscript AND at the end of the manuscript: Your ethics statement should only appear in the Methods section of your manuscript. If your ethics statement is written in any section besides the Methods, please delete it from any other section. | Ethics statement was removed except from the Methods section. |
| 5. In the online submission form, you indicated that data are available from the corresponding author upon request.  All PLOS journals now require all data underlying the findings described in their manuscript to be freely available to other researchers, either 1. In a public repository, 2. Within the manuscript itself, or 3. Uploaded as supplementary information.  This policy applies to all data except where public deposition would breach compliance with the protocol approved by your research ethics board. If your data cannot be made publicly available for ethical or legal reasons (e.g., public availability would compromise patient privacy), please explain your reasons on resubmission and your exemption request will be escalated for approval. | Data described in the manuscript is provided freely as supplementary files and the statement was amended to:  The datasets used and/or analyzed during the current study are available as supplementary information under the name S1_dataset. The survey instrument used is available as supplementary file S2_ Instrument. |

**Reviewer 1**

The manuscript presents a significant and timely study that evaluates physicians' knowledge, attitudes, and practices regarding complementary and alternative medicine (CAM) in the Jazan region of Saudi Arabia. Given the rising global interest in CAM, it is crucial for healthcare providers to have a solid understanding and positive outlook on these practices to ensure effective communication with patients and promote safe usage.

| Reviewer comments | Responses |
| --- | --- |
| The introduction lays a solid foundation regarding CAM and its global relevance. However, it would be beneficial for the authors to delve deeper into the specific reasons for conducting this study in the Jazan region. What particular characteristics or challenges in this area make the findings particularly valuable? | Thank you for your comment. We added the following sequence to address the comment:  “The Jazan region has unique geographic, cultural and healthcare landscape, since the population in this area is predominantly rural, with strong adherence to traditional and cultural practices [11, 12]. Additionally, there is evidence that the access to advanced healthcare services is limited in some local areas [13], which makes the choice of CAM a popular approach.” |
| 2. Methods:  1) The methods section is thorough and well-organized, detailing the study design, sampling methods, sample size calculation, and data analysis. Nonetheless, it would help to clarify the convenience sampling method used for physician recruitment and discuss any potential limitations this may introduce. | Thank you. We added the following sequence in “Sampling technique and sample size” subsection under the methods section:  “Such a sampling technique was used to allow efficient recruitment and inclusion of diverse healthcare settings. Efforts were made to reduce the potential selection bias (physicians who were more available or willing to participate may differ in their awareness) and enhance the representativeness of participants. This was done by recruiting participants from a variety of governorates and healthcare settings.” |
| 2)The authors mention that a validated survey was used for data collection. It would be useful to elaborate on the validation process of this survey and its reliability and validity specifically within the context of the Jazan region. | We already provided the following statement: “Given that the responses to attitude items were homogenous (i.e. the responses were consistently reported on a Likert scale), we were able to assess the internal consistency of the attitudes’ domain. Results of the reliability analysis showed a good level of internal consistency (Cronbach’s alpha = 0.812).”  The reliability of other domains was not calculated because they included responses with heterogenous responses (not consistent Likert scales) and multiple choice items.  The following paragraph was added:  "The survey instrument underwent a rigorous validation process specifically adapted for the Jazan context. Initially, the questionnaire was developed in English based on comprehensive literature review. **Content validity** was established through review by a panel of local experts (n=5) including CAM specialists, research methodologists, and practicing physicians from Jazan region who assessed item relevance, clarity, and cultural appropriateness. The Content Validity Index (CVI) was calculated, with items achieving a minimum CVI of 0.80 being retained. |
| 3. Results:  1) The results are clearly presented and well-structured. The use of both descriptive and inferential statistics effectively assesses the relationships between participants’ awareness, attitudes, and practices concerning CAM and their sociodemographic characteristics. However, a deeper exploration of the significance of these findings and their implications for practice and policy would enrich this section. | Thank you for the suggestion. We acknowledge the importance of discussing the implications of the findings in greater depth. To maintain the structure of the manuscript, we have added a sequence to the Discussion section, as the results section is intended solely to present the study's findings without interpretation or implications. The sequence is:  “The findings of the current study provide significant implications for practice and policy in the Jazan region and other areas with similar cultural patterns. The high levels of awareness, positive attitudes and the lack of formal pre-service training would all suggest the integration of CAM education into medical curricula and professional development programs to enhance CAM utilization in an effective and safe manner. Furthermore, tailored educational initiatives are required to address the specific needs of physicians’ groups, particularly considering the observed demographic differences in awareness and attitudes. Future policies should bridge these gaps to enhance CAM utilization based on robust evidence.” |
| 2) The manuscript states that the median attitude score was 23 out of 30 but does not provide a detailed breakdown of these scores across different demographic groups. Including this information would offer readers greater insight into the diversity of attitudes toward CAM among physicians. | Thank you for your comment. We added table 5 providing details about the breakdown of attitudes scores as suggested. |
| 4. Discussion:  1) The discussion is well-articulated and thoughtfully analyzes the findings. The authors effectively link the implications of their results to educational initiatives aimed at enhancing physicians’ competencies in CAM. That said, it would be advantageous to discuss potential barriers to incorporating CAM into clinical practice and propose specific strategies to address these obstacles. | We added the following paragraph to the discussion section to discuss potential barriers, with future recommendations:  “Importantly, the current study did not explicitly investigate barriers to CAM integration into local clinical practice. However, since a significant proportion of physicians (65.4%) agreed that medical practitioners should be educated in CAM use, a gap in formal education seems to be a considerable barrier. Additionally, the lack of structured training was an apparent barrier, since only 7.5% of the participants had received pre-service training in CAM. The relatively small percentage of physicians planning to apply CAM in the clinical practice (25.8%) may also reflect a lack of confidence in knowledge regarding CAM modalities. These findings are in agreement with other studies which suggest that healthcare professionals are hesitant to integrate CAM due to limited evidence-based knowledge, lack of educational resources and lack of institutional support [18, 19]. Future research should explore these barriers in details, potentially through mixed methods to assess the challenges faced by healthcare professionals in CAM integration into clinical practice.” |
| 2) The authors mention that the level of awareness of CAM aligns with findings from other regions in Saudi Arabia. However, a more detailed comparative analysis with those studies would strengthen the discussion, highlighting both similarities and differences in the results. | Thank you for your comment. We added the following paragraph to address the comparative analysis with other Saudi studies:  The level of awareness in the current study towards CAM (81.1%) aligns with the results from other regions in Saudi Arabia despite some variations. For example, a survey carried out in Tabuk region showed that a vast majority of residents (95.8%) were aware about CAM [7], indicating a relatively higher level of awareness than in Jazan. Similarly, the majority of primary healthcare physicians in Riyadh (88.9%) had some knowledge of CAM, including cupping, honey and herbal medicine as the most recognized practices [18]. CAM awareness was also high in Qassim region among physicians (77.1%) [19]. On the other hand, a lower awareness level was apparent in Madinah, where almost three-quarters of physicians (72.9%) acknowledged the need to gain knowledge about CAM [8]This underscores a significant desire for education and training, although the level of awareness seems satisfactory. Accordingly, targeted initiatives that provide effective educational materials to bridge gaps and support the competency of healthcare professionals in CAM practices are needed. The educational need is paramount given the prominent lack of pre-service training [18]. |
| 5. Limitations:  1) The authors briefly address the limitations of the study, such as the small sample size and the lack of an in-depth exploration of the reasons behind physicians' attitudes and practices. A more extensive discussion of how these limitations may affect the generalizability of the findings would be helpful. | We provided more details about the generalizability of findings in the following sequence:  “Although the sample under study was obtained from five regions in Jazan, the study settings remain limited to a specific geographical area, which might restrict the generalizability of findings to the other areas inside and outside Saudi Arabia. Indeed, the specific cultural and healthcare context of the Jazan region might have contributed to unique patterns of cultural perceptions of CAM and access to training opportunities, which are different to other areas. Future studies might be warranted in other regions including, urban regions and diverse healthcare systems to assess whether similar patterns and barriers to CAM integration are observed.” |
| 6. Data Availability:  1) The authors indicate that all relevant data are included within the manuscript and supporting information files. However, it would be beneficial to clarify whether the survey instrument used is available for other researchers to utilize or replicate the study. | We added the used survey in the supplementary material (S2_ Instrument). |
| Minor Comments:  1. A thorough proofreading of the manuscript is recommended to catch any grammatical, punctuation, or spelling errors | The manuscript has been edited by a professional proofreader. |
| 2. The authors should ensure consistency in formatting and citation style throughout the document. | The formatting and citation styles have been confirmed for consistency. |

Overall, this manuscript offers valuable insights into physicians’ knowledge, attitudes, and practices concerning CAM in the Jazan region of Saudi Arabia. With some revisions and clarifications, it holds great potential to contribute meaningfully to the field.

**Reviewer 2**

Thanks for your valuable investigation in terms on CAM. Some points mentioned below:

| Reviewer comments | Responses |
| --- | --- |
| The duration of sampling was 3 months which could be wider to earn more participant. Your involved 159 physicians, which might not be representative of the entire physician population in the Jazan region. | Thank you for your comment. The sampling duration of three months was sufficient to reach the target sample size of 159 physicians, as determined by the sample size calculation using the Rao soft sample size calculation formula.  Achieving this target ensures statistical reliability and validity for our study objectives. While a longer sampling period may have allowed for the inclusion of more participants, the obtained sample is representative of the physician population in Jazan due to the adherence to the calculated sample size and the inclusion of physicians from diverse healthcare settings (primary, secondary, and tertiary care facilities) across five governorates.  So, expanding the duration or increasing the sample size would not significantly alter the reliability of the results, as the calculated sample size already provides sufficient power to detect meaningful differences and trends. |
| Please give information about validity and reliability of your questionnaire | We provided the following statement:  The survey instrument underwent a rigorous validation process specifically adapted for the Jazan context. Initially, the questionnaire was developed in English based on comprehensive literature review. **Content validity** was established through review by a panel of local experts (n=5) including CAM specialists, research methodologists, and practicing physicians from Jazan region who assessed item relevance, clarity, and cultural appropriateness. The Content Validity Index (CVI) was calculated, with items achieving a minimum CVI of 0.80 being retained.  **Face validity** was established through pilot testing with 10 physicians from different healthcare facilities in Jazan who were not included in the final sample. The pilot testing assessed comprehension, cultural appropriateness, and time required for completion. Based on pilot feedback, minor modifications were made to improve clarity and cultural relevance of certain items. The internal consistency reliability was assessed using Cronbach's alpha, which was 0.81 |
| The study notes that only 25.8% of physicians plan to use CAM in practice, but it does not explore the barriers to integration in detail. Identifying and addressing these barriers could provide more actionable insights. | Although we did not explicitly assess the barriers via our questionnaire, we can conclude some barriers from some items. We added the following paragraph to the discussion section to discuss such a limitation, with future recommendations:  Importantly, the current study did not explicitly investigate barriers to CAM integration into local clinical practice. However, since a significant proportion of physicians (65.4%) agreed that medical practitioners should be educated in CAM use, a gap in formal education seems to be a considerable barrier. Additionally, the lack of structured training was an apparent barrier, since only 7.5% of the participants had received pre-service training in CAM. The relatively small percentage of physicians planning to apply CAM in the clinical practice (25.8%) may also reflect a lack of confidence in knowledge regarding CAM modalities. These findings are in agreement with other studies which suggest that healthcare professionals are hesitant to integrate CAM due to limited evidence-based knowledge, lack of educational resources and lack of institutional support [18, 19]. Future research should explore these barriers in details, potentially through mixed methods to assess the challenges faced by healthcare professionals in CAM integration into clinical practice. |
| Consider whether the findings can be generalized to other regions or healthcare systems. The cultural and healthcare context of the Jazan region might limit the applicability of the results elsewhere. | Thank you. We added the following sequence to the discussion section to address the comment:  “Although the sample under study was obtained from five regions in Jazan, the study settings remain limited to a specific geographical area, which might restrict the generalizability of findings to the other areas inside and outside Saudi Arabia. Indeed, the specific cultural and healthcare context of the Jazan region might have contributed to unique patterns of cultural perceptions of CAM and access to training opportunities, which are different to other areas. Future studies might be warranted in other regions including, urban regions and diverse healthcare systems to assess whether similar patterns and barriers to CAM integration are observed.” |
| You said that only 25.8% of physicians plan to use CAM in practice, but it does not explore the barriers to integration in detail. Identifying and addressing these barriers could provide more actionable insights. | Thank you for your comment. We addressed this comment above. |

**Reviewer 3**

| Reviewer comments | Responses |
| --- | --- |
| The title should be changed by adding “Alternative “ on the phrase complementary medicine i.e CAM | The title has been changed to “Bridging the Knowledge-Practice Gap: A Cross-sectional Survey Assessing Physician Knowledge, Attitude and Practice toward Complementary and Alternative Medicine” |
| The abstract needs to be written according to the journal guidelines. The statistics reported in the results section need improvement, e.g.; female physicians were not mentioned anywhere. | The abstract has been edited to be clearer and more adherent to the journal guidelines. We also clarified the proportions of female physicians. |
| Authors must provide references for most of the statements used in the manuscript. | We added multiple references to statements that required doing so. Thank you for your comment. |
| Authors should not generalise their findings to health practitioners while they only focus on physicians, as it can lead to misleading conclusions. | In the discussion section, we added some sequences to specifically focus our sequences on physicians rather than healthcare practitioners in general. |
| A sample size calculation formula should be indicated, as it ensures the study is statistically valid and reliable. | We added the used formulas for sample size calculation.  The sample size was calculated using a 95% confidence interval and a margin of error of 5% using the following formulas[21]:  $x=Z^{2}\times r\times\left( 100-r \right)$  $n=\frac{N\times x}{\left( N-1 \right)E^{2}+x}$  $E=\sqrt{\frac{\left( N-n \right) x}{n\times\left( N-1 \right)}}$  Where N is the population size (2104 physicians), r is the response distribution (87.5%), Z is the critical value for a confidence level of 95% (1.96). |
| The results section needs to be summarised. Not everything should be presented, especially tables. | Thank you for your comment. We amended the results section so that only significant associations were mentioned for all tables (1 to 8). For descriptive tables and figures, we only mentioned the categories with highest proportions as important results. |
| The discussion section and the conclusion section have to be improved. | The discussion section has been extensively edited, including the comparative analysis with other national and international studies, discussing the study limitations and future implications.  The conclusion was focused more and improved. |
| More comments and suggestions have been indicated in the manuscript as track changes. | All comments on the manuscript were addressed and changes in the manuscript have been marked as track changes |

**Reviewer 4**

The manuscript is good study exploring the KAP among Jazan physicians which is very important for easy integration of complementary medicine with current conventional medicine practices. Few comments are important to be addressed by the authors which are:

| Reviewer comments | Responses |
| --- | --- |
| The title; I think didn't address the content of the study, I suggest to be " Knowledge, Attitude and Practice toward Complementary Medicine Among Physicians: A Cross Sectional Study in Jazan” | Thank you for your suggestion. We edited the title to “Knowledge, Attitude and Practice toward Complementary and Alternative Medicine Among Physicians: A Cross-Sectional Study in Jazan” |
| Introduction:  Please add a description of the current situation of complementary medicine regulation in Saudi Arabia, you can use this published manuscript with the Title "A new official national regulations for complementary medicine practices in Saudi Arabia". And with the title "The present state of complementary medicine regulation in Saudi Arabia." | Thank you. We added the following sequence to the introduction section:  CAM regulation in Saudi Arabia is overseen by the National Center for Complementary and Alternative Medicine (NCCAM), which has been established under the Ministry of health in 2009 [7]. NCCAM is responsible for regulating CAM practices, licensing clinics and practitioners and enhancing public awareness. The 2019 NCCAM regulation represented a significant advancement in the governance of CAM practices, where only licensed practitioners are allowed to offer CAM services after passing standardized exams [7, 8]. NCCAM has the authority to add or suspend practices beyond five approved modalities (cupping therapy, naturopathy, acupuncture osteopathy, and chiropractic), a matter which reflects the country’s commitment to ensure the efficacy and safety of CAM [7]. |
| Results  Please do not repeat information which are presented in Tables and figures again in the Text.. you can just highlight the important findings | Thank you for your comment. We amended the results section so that only significant associations were mentioned for all tables (1 to 8). For descriptive tables and figures, we only mentioned the categories with highest proportions as important results. |
| Discussion:  Please add a section regrading the attitude of Medical students in Saudi Arabia towards complementary medicine espicially after implementation of complementary medicine curriculum with their medical studies, you have examples from Majmaa University and Taibah University. | We have added the following paragraph to address attitude of medical students in Saudi Arabia:  The findings of the present study have shed light into the importance of CAM integration into the medical curriculum to enhance the attitudes of medical students in Saudi Arabia. A study conducted at Majmaa University showed that students who received formal education were more likely to be confident to understand and counsel patients on CAM practices [28]. In another study carried out at King Saud University and Majmaa University [29], students were interested in learning about CAM, and a significant proportion of them supported the inclusion of CAM content in the medical curriculum. Additionally, although a small proportion of students had taken a course in CAM (15%), the educated students had positive attitudes towards traditional modalities [29]. |
| Add a paragraph regarding the importance of implementation of complementary medicine into medical curriculum. | We added the following paragraph to the discussion section to address the importance of CAM implementation into medical curriculum:  These results underline the significance of CAM incorporation into medical education to equip future physicians with the required skills and knowledge to address patients’ interests in CAM. As a consequence, formal CAM education in medical schools might address the aforementioned gap by fostering evidence-based understanding of CAM therapies, including their benefits and potential risks. CAM integration in medical education would not only enhance physicians’ competency, but also would promote safe and informed patient care. |

**Reviewer 5**

I liked the topic raised in the paper. The study has very interesting data.

| **Reviewer comments** | **Responses** |
| --- | --- |
| I believe that the problem of the population using different complementary medicines, some of which are prescribed by doctors, and the doctors themselves not having received adequate training for this, could be reinforced a little more (one paragraph). | Thank you. We added the following paragraph to the discussion section:  The current study highlighted a significant gap between physicians recommending or endorsing CAM practices and their lack of formal training related to CAM. Such a critical issue is apparent when physicians encounter patients who are actively using CAM or seeking CAM-related advice. In our study, while only 7.5% of physicians reported receiving a formal training, 37.7% of had recommended CAM to their patients. These findings highlight a critical disconnect between public CAM utilization and physician preparedness. This gap between popular practice and professional preparation raises concerns about patient safety and healthcare quality. When physicians lack formal training in CAM, they may be ill-equipped to guide patients who are already using or seeking advice about these treatments, potentially missing opportunities for integrated care or failing to prevent harmful interactions with conventional treatments."  . Similar observations were reported by another study in Riyadh [24], with multiple physicians expressing interest in discussing CAM practices with patients while only 8% of them had attended CAM-related training. As such, there is an urgent need to integrate structured CAM education into medical training programs in order to ensure that physicians are well-equipped with knowledge to prescribe CAM safely and effectively. |
| The acronym KAP was used twice in the text; the meaning should have been mentioned as soon as it appeared for the first time, at the end of the Introduction. | Thank you for your comment.  We added the acronym to the first instance in the following statement:  To address this critical issue, the current study aims to assess the knowledge, attitudes, and practices (KAP) of physicians regarding CAM in five governorates in Jazan. |
| I did not understand why non-English speakers were excluded. Why was this and whether it was possible to quantify how many there were? I believe that would be another relevant piece of information. | Non-English-speaking physicians were excluded from the study due to the language of the questionnaire, which was designed in English to align with the medium of instruction and communication commonly used in the healthcare system of Saudi Arabia. This decision was made to ensure the accuracy and validity of responses, as translation could introduce variability in interpretation and response quality. However, we recognize that not quantifying the number of non-English-speaking physicians limits the ability to assess their potential impact on the findings. While it was not feasible to retrospectively collect this data, we acknowledge this as a limitation of the study. Future research should consider bilingual or multilingual instruments to capture a more inclusive sample, ensuring representation of all physicians regardless of language proficiency. |
| I did not understand when they mentioned positive attitude and how this was measured. I was confused about whether it was about doctors recommending CAM to patients, applying CAM in their practices, participating in studies and research on CAM, encouraging patients to use CAM... | Non-English-speaking physicians were excluded from the study due to the language of the questionnaire, which was designed in English to align with the medium of instruction and communication commonly used in the healthcare system of Saudi Arabia. This decision was made to ensure the accuracy and validity of responses, as translation could introduce variability in interpretation and response quality. However, we recognize that not quantifying the number of non-English-speaking physicians limits the ability to assess their potential impact on the findings. While it was not feasible to retrospectively collect this data, we acknowledge this as a limitation of the study. We added the following sequence to the study limitations:  “Additionally, the exclusion of non-English speaking physicians due to the language of the survey. This might have resulted in the underrepresentation of specific physicians within the Jazan region, especially those who usually communicate with such a language. Future studies should consider validated, bilingual instruments to optimize representativeness and inclusitivity.” |

**Reviewer 6**

This study assessed physicians’ awareness, attitudes, and practices regarding complementary and alternative medicine (CAM) in Jazan through a cross-sectional survey. Based on data collection and basic analysis, the study found that although physicians exhibit high awareness and positive attitudes towards CAM, they lack formal training and practical experience. In the future, it is necessary to provide them with more training and guidance.

| Reviewer comments | Responses |
| --- | --- |
| Major  1.Have you considered potential subjectivity in the survey responses? If so, please explain how it was addressed. | We acknowledge the potential for subjectivity in survey responses. However, to minimize this, the survey was designed to ensure anonymity and confidentiality, which can encourage honest and unbiased responses. Additionally, the questionnaire included standardized, validated items to measure awareness, attitudes, and practices related to CAM, which reduces variability in interpretation and response. The Likert-scale format used for assessing attitudes was internally validated, achieving a Cronbach’s alpha of 0.812, indicating good reliability. While we recognize that some level of subjectivity may persist, these measures were implemented to enhance the validity and reliability of the collected data. |
| 2.In the Methods section, you mention that “visiting physicians or non-English speakers were excluded.” Please provide evidence supporting the reasonableness of this exclusion criterion. | Thank you for your comment. We have added the following sequence to the methods section:  The exclusion of visiting physicians was adopted due to their temporary service status, which might not actually reflect their typical practice and attitudes in the Jazan region compared to those with permanent services. Additionally, the use of an English-language questionnaire and subsequent exclusion of non-English speakers was methodologically necessary to maintain research quality and validity. This approach aligns with the current medical education and practice environment in Saudi Arabia, where English serves as the primary language for medical education, scientific literature, and professional communication. English proficiency is a standard requirement in our healthcare institutions, used in medical documentation, case discussions, and continuing education. The questionnaire contained specific CAM-related medical terminology that is standardly used in English, and using the original English version helped maintain the integrity of technical terms while avoiding potential meaning loss or alterations that could occur in translation. While we acknowledge this criterion was included in our methodology, it is worth noting that no participants were actually excluded from the study based on English language proficiency, as this reflects the standard language requirements for medical practice in our setting." |
| 3.In the Methods section, you state that “a score higher than the median was considered indicative of a positive attitude toward CAM.” If all scores are low, this approach would still categorize half of the participants as having a positive attitude. Is this method truly rigorous? | We acknowledge the limitation of using the median score to define a positive attitude, as it inherently categorizes half of the participants as having a positive attitude, regardless of whether the scores are objectively high or low. However, this approach was chosen due to the lack of standardized cutoff scores for interpreting attitudes toward CAM. The median provides a practical and internally consistent threshold for comparative analysis within the study population.  To address this potential limitation, the raw attitude scores (ranging from 6 to 30) and their distribution were also reported to provide additional context for interpreting the findings. The median attitude score of 23 observed in this study indicates a relatively positive orientation toward CAM overall, as it is closer to the upper end of the scale. We also analyzed the statistical differences in attitude scores (Table 5). |
| 4.In Table 4 of the Results section, while the p-value is <0.001, the difference between 3.3% and 8.5% is minimal, indicating that few physicians received pre-service training. Is this difference truly statistically significant? | While the difference between 3.9% and 8.5% regarding physicians’ awareness about CAM may appear minimal in absolute terms, the p-value of 0.009 indicates that the difference is statistically significant. This statistical significance reflects the reliability of the observed difference within the study sample, rather than the magnitude of the difference itself. The low percentage of physicians who received pre-service training (regardless of subgroup) underscores a critical gap in CAM education, even if the relative difference between groups is small.  We recognize the importance of interpreting statistical significance in the context of clinical or practical relevance and have clarified this in the discussion. |
| 5.Please analyze or explore the reasons that different populations may have varying attitudes and levels of understanding toward CAM. | Thank you. We added the following paragraph to the discussion section to address your comment:  Our study demonstrated significant variations in awareness and attitudes among physicians, which might be attributed to broader factors affecting different populations. Cultural beliefs and societal norms are crucial determinants of CAM perceptions, particularly in Saudi Arabia. This is evident for distinct practices, like cupping therapy and prophetic medicine [21]. Furthermore, the lack of structured training and education can influence participants’ attitudes and awareness, where limited exposure to CAM during medical education might decrease the level of confidence and understanding [32]. These observations might explain the reasons of having varying attitudes and levels of understanding of CAM, and these would help tailor effective solutions to increase physicians’ attitudes. |
| 6.In the Background and Introduction sections, provide conclusive evidence demonstrating that CAM is indeed effective in treating diseases.Relative reference such as PMID: 34896048 and PMID: 36939781 could be cited to introduce current application and research on CAM | Thank you. We added the following paragraph to address the comment:  Actually, CAM therapies have been increasingly supported by scientific evidence worldwide demonstrating the effectiveness of these therapies in reducing pain, managing chronic illnesses and enhancing overall well-being [6]. In Arab and Islamic regions, CAM therapies represent an integral part of religious and cultural traditions. Black seed and honey have shown promising outcomes due to their antimicrobial, anti-inflammatory and immune-boosting properties [6]. Cupping therapy have also demonstrated effective results in alleviating musculoskeletal pain and improving the quality of life of patients with chronic pain [7]. Ruqyah (spiritual healing) and honey had also important roles in improving mental health and reducing stress, and herbal medicine remedies are increasingly utilized to reduce symptoms and improving the quality of life of patients with mental health conditions [6, 7]. |
| 7.How do you propose improving CAM education and training? | Thank you. We added the following paragraph:  From a practical perspective, CAM education and training can be improved via integrating CAM educational materials into undergraduate medical curricula and implementing continuing medical education programs and professional development opportunities. These scientific materials should focus on evidence-based CAM to assist in improving physicians’ practices based on reliable therapies [32]. Such efforts would be ideally offered via structured training modules, practical exposure and providing consistent updates on novel evidence to ensure that physicians are well-prepared to use CAM effectively and safely. |
| 8.Given the cultural and economic differences between this region and others, have you considered unique factors or alternative approaches to implementing changes? | Thank you for your comment. We added the following sequence:  Indeed, the unique cultural and economic context of the Jazan region and similar regions should be considered when implementing changes in CAM education and integration. The widespread use of honey, Ruqyah and Hijama can be exploited to support the acceptance of CAM education and training. The incorporation of these cultural beliefs and evidence-based CAM therapies into training programs can ultimately lead to an improved engagement. Economically, the integration of CAM practices into public healthcare offerings and subsidizing CAM services can reduce their costs and promote equitable access to CAM, especially in rural and unsupported areas. |
| 9.Are you certain that South Korea has the highest rate of CAM usage in Asia, rather than China? This is somewhat surprising. | We searched in the literature for nationally-representative studies conducted in China, but we could not find nation-wide studies. The highest CAM utilization rates were found in Singapore and South Korea. |
| 10. All tables currently display detailed data. Consider presenting only the most critical data in the tables**, with additional data included in the supplementary materials.** | Thank you for your suggestion.  We amended the results section so that only significant associations were mentioned for all tables (1 to 8). For descriptive tables and figures, we only mentioned the categories with highest proportions as important results.  While we acknowledge the utility of supplementary materials for extensive datasets, in this case, condensing the information could obscure important aspects of the results and detract from the study's overall impact. |
| 11. Please provide a legend for each graph. | Thank you for your observation. We would like to clarify that legends have been provided for all figures in the manuscript (below each figure). |
| 12. Indicate how you conducted random sampling across the five regions. | We apologize for any confusion and need to clarify our sampling methodology. Our study actually employed convenience sampling rather than random sampling. Participants were recruited from healthcare facilities across the five regions of Jazan based on accessibility and willingness to participate. While this sampling approach allowed us to achieve a high response rate and include physicians from various healthcare settings, we acknowledge this as a limitation of our study. We have revised any potentially confusing statements in the manuscript to clearly indicate that convenience sampling was employed in our study. |
| 13.Attach the complete questionnaire in the supplementary materials. | We provided the questionnaire as supplementary file: S2_Instrument. |
| 14.Many of the references are either outdated or from relatively lesser-known journals. Consider updating them as appropriate. | Thank you for your feedback. We have carefully reviewed the references cited in the manuscript and ensured that they include studies from authentic and reputable sources. |
| 15.The study only includes two figures. Consider adding more visual representations of the data. | Thank you for your comment. We added a new figure as per your suggestion (Figure 3). |
| 16.In the Conclusion section on the second page, there are two consecutive commas—please correct this. | After reviewing the updated manuscript, the consecutive commas in the conclusion section were not found. If this issue persists or is identified in another context, please highlight the specific sentence for further correction. |
